# The relationship between physical activity level and balance parameters, muscle strength, fear of falling in patients with hypertension

**DOI:** 10.1097/MD.0000000000036495

**Published:** 2023-12-01

**Authors:** Necati Özler, Mehtap Malkoç, Ender Angin

**Affiliations:** a European University of Lefke, Faculty of Health Sciences, Departments of Physical Therapy and Rehabilitation, Lefke, Turkey; b Eastern Mediterranean University, Faculty of Health Sciences, Department of Physiotherapy and Rehabilitation, Famagusta, Turkey.

**Keywords:** balance, Fullerton’s Advanced Balance Scale, hypertension, physical activity level

## Abstract

The number of studies investigating the role of physical activity and exercise in hypertension (HT) patients is insufficient in the literature, and reports evaluating the relationship between HT, physical activity, and balance are lacking. This study aims to examine the relationship between physical activity levels and balance parameters, muscle strength, and fear of falling in patients with HT. 78 subjects with HT participated in this study. Demographic and clinical characteristics of all participants were recorded. Blood pressure was evaluated using a sphygmomanometer, physical activity level was assessed using a SenseWear Armband, fear of falling was assessed using the Fall Efficacy Scale, balance was assessed using the Fullerton Advanced Balance Scale, and muscle strength was evaluated using a digital handheld dynamometer. All 78 subjects completed the study as planned. The average age of participants was 57.75 ± 5.82, the mean systolic blood pressure was 133 ± 5.73, and the diastolic blood pressure was 84 ± 6.78. 34.2% of participants used angiotensin-converting enzyme inhibitors, 38% used beta blockers, and 26% used diuretic drugs. A positive correlation between physical activity and balance scores of individuals with HT was found (*P* < .005). It was also found that low muscle strength was associated with balance and risk of falling (*P* < .005). There is a positive correlation between decreased physical activity levels and balance in participants with HT. The results suggest that people with HT who have poor balance also have decreased muscle strength against gravity, such as in the quadriceps femoris and gluteus maximus. Overall, we recommend that patients with HT should improve their physical activity levels.

## 1. Introduction

Hypertension (HT) is the main condition that results in the highest mortality rate in the world.^[1]^ A systolic blood pressure of ≥ 140 mm Hg and a diastolic blood pressure of ≥ 90 mm Hg is known as HT. It is an important problem that increases the prevalence of cardiovascular diseases and causes disabilities.^[1,2]^ The studies show that physical activity can be used to prevent the risks of HT. It is known that people with arterial HT have significantly reduced physical capacity HT is frequently accompanied by symptoms such as headache, blurring of vision, tachycardia, thoracic pain, shortness of breath, weakness of limbs, and swollen ankles. These symptoms influence anatomical or functional alterations in people, which ultimately affect postural balance.^[2]^

The benefits of a physically active lifestyle have been well documented in the literature.^[3]^ These benefits include high levels of cardiovascular, skeletal, and metabolic muscle functions, people with HT are aware of the benefits of physical activity, but only a few of them engage in regular physical activity.^[4]^ Some studies have reported a possible association between HT and low physical activity. Theoretically, high blood pressure levels can damage arteries responsible for transporting blood to the brain, thereby limiting blood flow to the brain areas responsible for muscle contraction. Also, studies have shown that oxygen uptake is lower in hypertensive patients.^[2]^

Physical activity is known to have multiple benefits and is recommended to reduce the risk of falling.^[5]^ Few studies have examined the relationship between amounts of physical activity and postural balance, or assessed differences in amounts of physical activity categorized by intensity utilizing accelerometers in hypertensive patients. Fear of falling has been variously defined including concern that normal activities cannot be performed without falling, lack of confidence in maintaining balance during normal activities, and being frightened of falling.^[5]^

Physically inactive patients have less physiological reserves; therefore, functional fitness is limited, and a greater proneness to dependency often occurs.^[6]^ Limitations in physical activity decrease aerobic endurance which affects the ability to perform daily activities, such as walking long distances and climbing stairs.^[7]^ Physical activity and exercise, both aerobic and anaerobic, have favorable effects in the primary and secondary prevention of HT.^[8]^

Although there are a few studies in the literature exploring the role of physical activity and exercise in HT patients and assessing balance and fall risk in these individuals, reports evaluating the correlation between HT, physical activity and balance are missing. This clearly emphasizes the need for further studies in the field. In this regard, the current study aims to assess the relationship between physical activity levels, muscle strength, fall history, and balance parameters in persons diagnosed with HT.

## 2. Materials and methods

### 2.1. Study design

This cross-sectional study was conducted between January and November 2022. The study was approved by the Health Ethics Sub-Committee of Scientific Research and Publication Committee of Eastern Mediterranean University (December 12, 2021 and with the decision number 2022/0028). A written consent was obtained from all the participants. In addition, we obtained a clinical trial number (NCT05187702). To eliminate bias in our study, each set of data was assessed 3 times, and the averages were recorded. All assessments were conducted by the same individual in the same environment using identical equipment. Subsequently, all results were subjected to quantitative evaluation and statistical analysis.

### 2.2. Participants

The study was carried out with 78 participants, who were diagnosed with hypertension according to the criteria of the European Society of Hypertension/European Society of Cardiology at the Nicosia State Hospital, Polyclinics of Internal Medicine, and Cardiology.^[9]^ “Aerobic versus isometric handgrip exercise in hypertension: a randomized controlled trial” (https://doi.org/10.1097/HJH.0000000000001445) was taken as a reference to determine the sample size and power before assessing the individuals in the study, and it was determined that the effect size for the difference in SDB values between groups was large. Accordingly, f = 0.40 and 95% (1–β = 0.95) at α = 0.05 level. The sample size of the study was calculated using the G* Power software (version 3.1.9.2).

#### 2.2.1. Inclusion and exclusion criteria.

**Figure 1.**
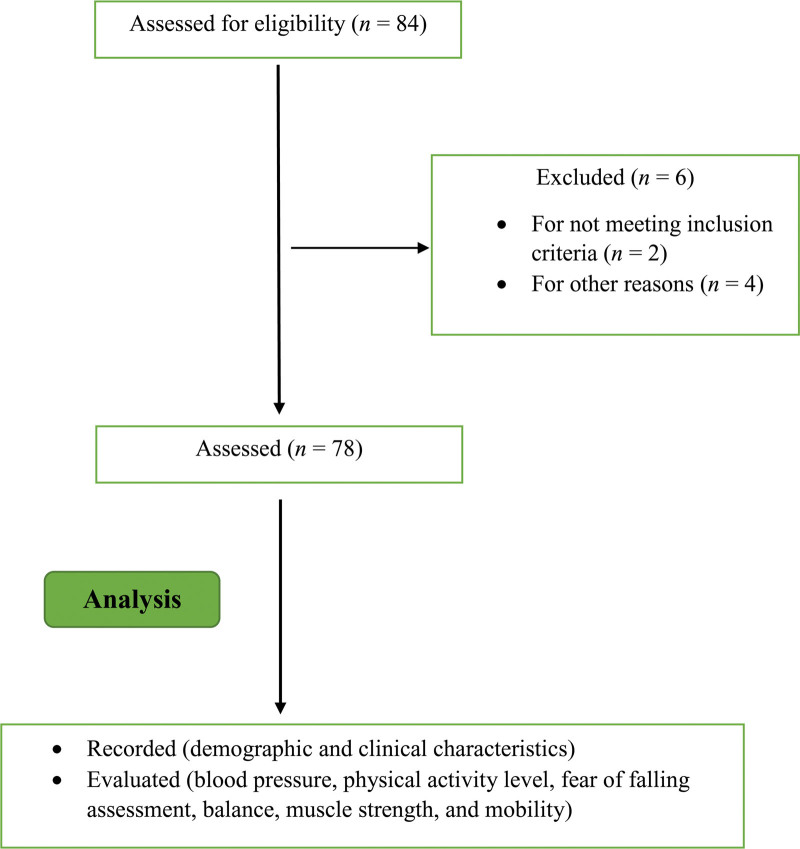
Participant flowchart according to STROBE. Out of 84 eligible subjects with hypertension, 6 were excluded for not meeting inclusion criteria, declining to participate, or other reasons. Demographic and clinical characteristics of the remaining 78 participants were recorded. Blood pressure, physical activity level, fear of falling assessment, balance, muscle strength, and mobility were evaluated.

Our inclusion criteria consisted of participants diagnosed with HT (for at least 6 months), aged between 45 and 65 years, independent in activities of daily living, capable of walking 400 m without assistance, not having experienced a 5% or greater loss of body weight in the preceding 3 months, and maintaining the same prescribed dosage by the same doctor. Those with unmanageable blood pressure, neurological diseases, chronic conditions like kidney failure and cancer, and those who faced impediments to exercise or were using multiple drugs (more than 5 medicines) were excluded from the study. The flowchart for all participants is shown in Figure 1.

#### 2.2.2. Outcome measures.

Demographic and clinical characteristics of all participants were recorded. Blood pressure, physical activity level, fear of falling assessment, balance, muscle strength, and mobility were evaluated.

A sphygmomanometer was used to assess the resting blood pressure in the sitting position. The blood pressure measurement protocol was based on 3 sequential measurements by using a sphygmomanometer, 1 minute apart between measurements, with the individuals in a sitting position. The first blood pressure measurement was performed after 30 minutes of resting in a sitting position. The same cuff-based device was used for measuring the blood pressure of all the participants, and the device was calibrated prior to the study as instructed before.^[9]^

Physical activity levels and energy consumption were measured for 3 days (72 hours) during daily activities with the SenseWear Armband. Before starting this protocol, each participant’s age, height, weight, dominant hand, date of birth, and smoking habits were recorded. The device software used step count and energy consumption values calculated from the accelerometer, physiological sensors, and demographic information developed by the manufacturer. After recording the personal information of the participants into the device, the “SenseWear Pro3” Armband was placed on the left triceps muscle at the midpoint of the acromion and olecranon. Participants were asked to take it off only for a maximum of 1 hour to take a shower, and wear it again throughout the day. As a result, total energy consumption, number of steps, sleep time, and rest periods were calculated.^[10]^

The fear of falling was assessed using the Fall Efficacy Scale. This scale comprises questions rated on a scale from 1 to 10, where 1 signifies “very confident” and 10 signifies “I have no trust at all” regarding the daily functions of the participants. The Turkish version of the scale’s validity and reliability test was conducted by Ağircan et al in 2009.^[11]^

Balance was assessed using Fullerton’s Advanced Balance Scale (FAB-T). This scale consists of 10 questions and is designed to evaluate balance, with a total possible score of 40 points. A score below 25 points suggests a high risk of falling. The Turkish reliability and validity study for this scale was conducted by İyigün et al in May 2020^[12]^ This scale is significant as it assesses static, dynamic, and reactive balance.^[12]^

We used the Single Leg Standing Test for static balance assessment. Participants were asked to stand on 1 leg as much as possible by pulling their hips in front of the body. If they could stop for 30 seconds, the test would be terminated. If participants stand for less than 10 seconds, there is a risk of balance disorder, and if they can stand for less than 5 seconds, there is a risk of falling.^[13]^

Muscle strength was assessed with a digital handheld dynamometer. Subjects were asked to contract their muscles for 5 seconds for each test. The tests were repeated 3 times, and the best value was recorded in kilograms (kg). To gain muscle recovery and reduce fatigue, a break of 15 seconds was given between repetitions and 2 minutes between movements.^[14]^

Mobility was assessed with the Time Up and Go Test and Sit to Stand Test. The participants completed the Time Up and Go Test in a neutral position with eyes open, hands on hips, bare feet, and hips in a neutral position. The time started as soon as the foot was lifted off the ground and was recorded in seconds. Three attempts were made for each foot, and the best value was recorded.^[15]^ During the Sit to Stand Test, participants were asked to sit in a chair with a back support in front of the body, then fold their hands in front of the body and stand up. During the test, the repetition of participants was recorded within 30 seconds. A score of < 10 indicated lower extremity weakness.^[16]^

### 2.3. Statistical analysis

Statistical analyses were performed using SPSS 20.0 software (IBM, Chicago, IL). Frequency analysis was used to determine the introductory characteristics of the participants included in the study. The fit of the data to the normal distribution was tested using the Kolmogorov–Smirnov and Shapiro–Wilk tests. We used non-parametric assumption tests and Spearman correlations to determine the relation between the variables analysis, as they were not suitable for normal application of our data. The data that support the findings of this study are available from the corresponding author [N.Ö.] upon reasonable request.

## 3. Results

There were 84 eligible subjects with HT, of which 6 were excluded for not meeting the inclusion criteria, declining to participate, or other reasons. The socio-demographic information of the patients is presented in Table 1.

**Table 1:** Physical and demographic characteristics of the cases.

N = 78	95% CI Mean					
	Median	Upper	Lower	IQR	Min	Max
Age (years)	55.5	59.05	56.46	8.75	46	65
Height (cm)	1.65	1.69	1.64	0.17	150	188
Body weight (kg)	76.00	80.65	74.61	22.50	58	110
BMI (kg/m^2^)	28.32	29.01	27.14	3.40	20.75	42.97
SBP (mm Hg)	133	142	125	5	120	145
DBP (mm Hg)	85	86	76	6	70	100
ACH inhibitors (n (%))	27 (34.2)					
Beta blockers (n (%))	30 (38)					
Diuretics (n (%))	21 (26)					
Gender (F/M) n (%)	44/19 (56.4/43.6)					

BMI = body mass index, F/M = female/male, IQR= quartile range.

The median values of number of falls, FAB-T score, Single Standing Test result (in seconds), Timed Up and Go Test score and Timed Up and Sit Test score were 3, 15.5, 12, 15, 8.5 respectively. Daily energy consumption (in kcal/day) was 354. Muscle strength data is presented in Table 2.

**Table 2:** Balance parameters, number of falls, daily energy consumption, daily total steps, daily rest time and muscle strength of the cases.

N = 78	95% CI Mean					
	Median	Upper	Lower	IQR	Min	Max
FAB-T	15.5	16	15	4	9	34
SST (min)	12	15	12	9	2	48
TUG (min)	15	15	13	6	6	30
TUST (min)	8,5	10	8	5	3	22
Number of falls (n)	3	3	2	1	1	4
Fall efficacy scale	39	39	36	5	15	52
Daily energy consumption (kcal/d)	354	390	354	115	245	536
Daily total steps (steps/d)	3781	4127	3753	1244	2536	5878
Daily rest time (hr/d)	5.4	5	5	2	3	9
Quadriceps strength (kg)	4.4	6	4	2	12	18
Gluteus maximus strength (kg)	5.9	6	6	2	6	11
Abdominal muscles (kg)	6.6	7	6	3	7	15
Back extensors (kg)	5.5	5	6	3	7	14

FAB-T = Fullerton Advanced Balance Scale, IQR= quartile range, SST = Single Stance Test, TUG = Timed Up and Go Test, TUST = Timed Up and Sit Test.

Relationships between FAB-T, daily energy consumption, muscle strength, and balance parameters of the cases are given in Table 3. Those less than 0.29 represent a low correlation. Correlation values between 0.3 and 0.7 represent medium correlation, and those greater than 0.71 represent high correlation.

**Table 3:** The Relationships between, daily energy consumption, FAB-T, muscle strength and balance parameters of the cases.

N = 78	FAB-T		SST		TUG		FES		STS	
	*R*	*P*	*R*	*P*	*R*	*P*	*R*	*P*	*R*	*P*
DEC	**0.342** **	**.002**	0.090	.433	**−0.344** **	**.002**	−0.153	.180	**0.275** *	**.015**
DTS	−0.017	.881	−0.103	.367	−0.190	.095	−0.034	.767	0.129	.259
DRT	0.041	.721	−0.024	.835	−0.016	888	0.194	.089	−0.192	.092
QS	**0.401** **	**.000**	**0.322** **	**.004**	0.004	.079	−0.002	.984	**0.383** **	.001
GMS	**0.504** **	**.000**	**0.240** *	**.035**	−0.212	.063	−0.061	.593	0.065	570
AMS	0.007	.954	0.032	.782	0.242*	.033	**−0.360** **	**.001**	−0.043	706
BES	0.183	.109	0.012	.919	0.082	.474	**−0.438** **	**.000**	−0.123	282

AMS= abdominal muscles strength, BES= back extensors strength, DEC = Daily Energy Consumption (kCal/d), DRT=Daily Rest Time (hr/d), DTS= Daily Total Steps (steps/d), FAB-T = Fullerton Advanced Balance Scale, FES= Fall Effica2cy Scale, GMS= Gluteus Maximus Strength, QS= Quadriceps Strength, SST = Single Stance Test, STS= Sit to Stand Test, TST = Total Sleep Time per Day (hour/day), TUG = Timed Up and Go Test.

* denotes correlation.

** denotes high correlation.

## 4. Discussion

This study was conducted to examine the relationship between physical activity level and balance parameters, mobility, muscle strength, and fear of falling in participants with a diagnosis of HT for at least 6 months. The results of this study showed that physical activity level was associated with balance. HT, a key risk factor for cardiovascular diseases, is the leading cause of disability. Over the past 4 to 5 decades, accumulated data have yielded consistent findings on protective effects of physical activity in the prevention of HT. Studies have shown that chronically elevated blood pressure is associated with mild deterioration in reaction time and physical performance and that the effects of high blood pressure on motor functions increase with age.^[12]^ While the mechanisms responsible for these observed problems in participants with HT are not yet fully understood, it is suggested that these issues may arise from reduced cerebral blood flow, metabolic changes, structural alterations in the cerebrum, or dysfunctions in the sympathetic nervous system.^[17]^

High arterial blood pressure, also known as HT, is a significant risk factor for the global burden of diseases and mortality.^[18]^ The effect of arterial HT on organ damage has been extensively studied. Studies have also reported that physical activity and exercise capacity are notably reduced in individuals with arterial HT.^[19]^ The lack of physical activity is a major risk factor for morbidity and mortality. Although the benefits of physical activity and an active lifestyle, including blood pressure control, are known to prevent cardiometabolic diseases, the global population struggles to implement it successfully. To reduce the risk of HT, promoting physical activity and fitness should be encouraged.^[19,20]^

There are few studies reporting that the motor unit firing rate in hypertensive participants is slower than in normotensive participants.^[20,21]^ In addition, when the lower extremities and upper extremities were compared in terms of reaction time, it was stated that the reaction time was slower due to the slower nerve conduction velocity in the lower extremities and the longer nerve trace.^[21]^ Studies have shown that individuals diagnosed with HT often have poor balance control.^[21]^ In addition to these symptoms, it has been reported that the effects of HT on the arteries may also adversely affect the cerebellum and cochleo-vestibular centers in the central nervous system.^[22]^ Hausdorff et al^[18]^ reported that HT not only increases the risks of cardiovascular disease, but also has adverse effects on balance and gait. In another study, Abate et al^[21]^ reported that dizziness and vertigo were more common in individuals with HT, and postural instability was more common, but there was no difference in performance compared to normotensives. In another study conducted with elderly individuals, Acar et al^[23]^ compared the balances of hypertensive individuals and individuals with normal blood pressure on a moving and inactive ground. As a result, they could not find any significant difference between the 2 groups and suggested that this should be done with a larger population. In the present study, we evaluated the balances of individuals with HT both dynamically and statically. In parallel with the literature, we also found that the balance scores of participants with HT were low (<24), and the scores of our dynamic tests, the timed up-and-go and sit-and-stand tests, were also decreased.

There are several studies showing that chronic diseases such as metabolic syndrome, diabetes, and HT cause energy metabolism disorders in the musculoskeletal system.^[24]^ Arterial HT has been demonstrated to accelerate sarcopenia, especially due to its effects on muscle circulation.^[25]^ In this sense, it is obvious that the muscles with large circulation, such as the trunk and lower extremity muscles, weaken due to this picture, which is especially seen in the antigravity muscles. In another study, Blanchard et al^[26]^ reported that high blood pressure would affect muscle metabolism compared to normal blood pressure. In addition, Dipla et al^[27]^ showed that the muscle structure of individuals with HT is affected by decreased muscle oxygenation due to mitochondrial dysfunction and impaired vasodilation. As a result, it is known that reduced antigravity affects muscle mass and balance, which in turn indirectly impacts the level of physical activity. Therefore, the relationship we found between physical activity level and other parameters reaffirms that higher physical activity levels lead to better balance, particularly in individuals with HT. In this study, it is observed that the timed get-sit test takes longer for individuals with HT who have low levels of physical activity, indicating that those who are less physically active find it more challenging to complete the test.

In this study, we evaluated antigravity muscle strength, considering that muscle strength would also be affected in participants with HT. We evaluated the gluteus maximus and quadriceps femoris muscles in the lower extremities, and the abdominal and back extensor muscles in the trunk. Our findings showed that individuals with HT with good lower extremity thigh circumference muscle strength had a better balance score, but individuals with HT with weak trunk muscles had a higher risk of falling. Muscle strength weakened by the decrease in physical activity is also associated with the balance of individuals with HT. Accordingly, the blood pressure is a potential risk factor that may lower postural stability.

The relationship between falls and chronic diseases was emphasized in different studies.^[28]^ In these studies, the existence of diseases that cause deterioration of muscle metabolism and affect the neural system is emphasized, especially among the factors that increase the risk of falling.^[28]^ It is seen that the reduction of reaction time, especially in the lower extremities, is of great importance in daily living activities in terms of balance problems and the risk of falling.

Reasons such as reduced reaction time, interference of balance centers due to impaired cerebral circulation, and drug use may cause individuals with HT to experience balance problems. Some studies conducted to date have shown that there is no significant difference in static balance measurements between HT and normotensive individuals.^[21]^ but it should be noted that during activities of daily living, dynamic and reactionary balance is more important in terms of functionality as well as static balance.

In this study, we evaluated the balance of participants with HT both statically and dynamically, and also the risk of falling using the Fall Efficacy Scale. We found that the median balance scores in our study were 15.5 in individuals with HT. Since this value was below the cutoff value of 24, the static, dynamic, and reactive parameters of individuals with HT were considered low. Moreover, we also showed that this condition is associated with important antigravity muscle forces such as quadriceps femoris and gluteus maximus. These findings show that participants with good lower extremity thigh circumference muscle strength have better balance, but individuals with weak trunk muscles have a higher risk of falling.

To minimize bias in our study, the dosage of blood pressure medication administered to the patients was standardized by the same physician, as specified in our inclusion criteria. However, it is important to note a limitation of this study. Dizziness and balance issues may potentially arise as side effects of these medications. However, it was not within the scope of our study to specifically investigate the effects of these drugs, hence we cannot provide definitive information on this matter.

## 5. Conclusion

In conclusion, significant relationships between physical activity and balance were observed in participants with HT. Additionally, the correlations with antigravity muscle strength are noteworthy. Based on these findings, physiotherapists focusing on balance and strength-building programs for large muscle groups while planning the treatment of individuals with HT can enhance the effectiveness of HT rehabilitation and contribute to the field of physiotherapy. It is also recommended that individuals with HT become more active and strive to improve their physical activity levels.

## Author contributions

**Formal analysis:** Necati Özler.

**Investigation:** Necati Özler.

**Methodology:** Necati Özler, Mehtap Malkoç.

**Resources:** Necati Özler.

**Supervision:** Mehtap Malkoç, Ender Angin.

**Writing – original draft:** Necati Özler.

**Writing – review & editing:** Mehtap Malkoç, Ender Angin.

## References

[1] Poulter NR Prabhakaran D Caulfield M. Hypertension. Lancet. 2015; 386:801–12. 25832858 10.1016/S0140-6736(14)61468-9

[2] Laffin LJ Bakris GL. Hypertension and new treatment approaches targeting the sympathetic nervous system. Curr Opin Pharmacol. 2015;21:20–4. 25541034 10.1016/j.coph.2014.12.006

[3] Warburton DER Bredin SSD Charlesworth SA. Evidence-based risk recommendations for best practices in the training of qualified exercise professionals working with clinical populations. Appl Physiol Nutr Metab. 2011;36:S232–65. 21800944 10.1139/h11-054

[4] Fletcher BJ Gulanick M Braun LT. Physical activity and exercise for elders with cardiovascular disease. Medsurg Nurs. 2005;14:101–9; quiz 110. 15916265

[5] Guirguis-Blake JM Michael YL Perdue LA. Interventions to Prevent Falls in Community-Dwelling Older Adults: A Systematic Review for the U.S. Preventive Services Task Force. Rockville, MD: Agency for Healthcare Research and Quality (US); 2018. (Evidence Synthesis, No. 159). Available at: https://www.ncbi.nlm.nih.gov/books/NBK525700/30234932

[6] Volschenk A. The Association Between Physical Activity, Functional Fitness, and Balance in Senior Citizens [dissertation]. North-West University; 2011.

[7] Pedrosa R Holanda G. Correlation between the walk, 2-minute step, and TUG tests among hypertensive older women. Braz J Phys Ther. 2009;13:252–6.

[8] Caminiti G Iellamo F Mancuso A. Effects of 12 weeks of aerobic versus combined aerobic plus resistance exercise training on short-term blood pressure variability in patients with hypertension. J Appl Physiol. 2021;130:1085–92. 33630677 10.1152/japplphysiol.00910.2020

[9] Mancia G Fagard R Narkiewicz K.; Task Force for the Management of Arterial Hypertension of the European Society of Hypertension and the European Society of Cardiology. 2013 ESH/ESC practice guidelines for the management of arterial hypertension: ESH-ESC the task force for the management of arterial hypertension of the European Society of Hypertension (ESH) and of the European Society of Cardiology (ESC). Blood Press. 2014;23:3–16.24359485

[10] Çetin C. Metabolik holter ile fizik tedavi ve rehabilitasyon bölümü öğrencilerinin günlük fiziksel aktivitesinin ölçülmesi. Ankara Üniversitesi Tip Fakültesi Mecmuasi 2008;61:196–201.

[11] Tinetti ME Williams TF Mayewski R. Fall risk index for elderly patients based on number of chronic disabilities. Am J Med. 1986;80:429–34. 3953620 10.1016/0002-9343(86)90717-5

[12] Iyigun G Kirmizigil B Angin E. The reliability and validity of the Turkish version of Fullerton Advanced Balance (FAB-T) scale. Arch Gerontol Geriatr. 2018;78:38–44. 10.1016/j.archger.2018.05.022 29886283

[13] Vellas BJ Wayne SJ Romero L. One-leg balance is an important predictor of injurious falls in older persons. J Am Geriatr Soc. 1997;45:735–8. 9180669 10.1111/j.1532-5415.1997.tb01479.x

[14] Hürer C Angin E Tüzün EH. Effectiveness of clinical Pilates and home exercises in sagittal cervical disorientation: randomized controlled study. J Comp Eff Res. 2021;10:365–80. 33706543 10.2217/cer-2020-0186

[15] Bohannon RW. Reference values for the timed up and go test: a descriptive meta-analysis. J Geriatr Phys Ther. 2006;29:64–8. 16914068 10.1519/00139143-200608000-00004

[16] Jones CJ Rikli RE Beam WC. A 30-s chair-stand test as a measure of lower body strength in community-residing older adults. Res Q Exerc Sport. 1999;70:113–9. 10380242 10.1080/02701367.1999.10608028

[17] Pirodda A Brandolini C Modugno C. Hypotension associated with autonomic dysfunction: a possible cause of vertigo? Med Hypotheses. 2004;63:1086–1086. 15504583 10.1016/j.mehy.2004.07.004

[18] Hausdorff JM Herman T Baltadjieva R. Balance and gait in older adults with systemic hypertension. Am J Cardiol. 2003;91:643–5. 12615286 10.1016/s0002-9149(02)03332-5

[19] Cornelissen VA Fagard RH Coeckelberghs E. Impact of resistance training on blood pressure and other cardiovascular risk factors: a meta-analysis of randomized, controlled trials. Hypertension. 2011;58:950–8. 21896934 10.1161/HYPERTENSIONAHA.111.177071

[20] Moraes WMD Souza PRM Pinheiro MHNP. Exercise training program based on minimum weekly frequencies: effects on blood pressure and physical fitness in elderly hypertensive patients. Braz J Phys Ther. 2012;16:114–21. 10.1590/s1413-35552012005000013 22481693

[21] Abate M Di Iorio A Pini B. Effects of hypertension on balance assessed by computerized posturography in the elderly. Arch Gerontol Geriatr. 2009;49:113–7. 10.1016/j.archger.2008.05.008 18619684

[22] Borghi C Modugno GC Brandolini C. Is tinnitus useful in early detection of incoming heart decompensation?. Med Hypotheses. 2006;67:437–9. 16624499 10.1016/j.mehy.2006.01.061

[23] Acar S Demirbüken I Algun C. Is hypertension a risk factor for poor balance control in elderly adults?. J Phys Ther Sci. 2015;27:901–4. 25931755 10.1589/jpts.27.901 PMC4395739

[24] Wasserman K Whipp BJ. Exercise physiology in health and disease. Am Rev Respir Dis. 1975;112:219–49. 239617 10.1164/arrd.1975.112.2.219

[25] Evans WJ Campbell WW. Sarcopenia and age-related changes in body composition and functional capacity. J Nutr. 1993;123:465–8. 8429405 10.1093/jn/123.suppl_2.465

[26] Blanchard AR Taylor BA Thompson PD. The influence of resting blood pressure on muscle strength in healthy adults: resting blood pressure and muscle strength. Blood Press Monit. 2018;23:185–90. 29738358 10.1097/MBP.0000000000000325 PMC6035107

[27] Dipla K Triantafyllou A Koletsos N. Impaired muscle oxygenation and elevated exercise blood pressure in hypertensive patients: links with vascular stiffness. Hypertension. 2017;70:444–51. 28607132 10.1161/HYPERTENSIONAHA.117.09558

[28] De Rekeneire N Visser M Peila R. Is a fall just a fall: correlates of falling in healthy older persons The Health, Aging and Body Composition Study. J Am Geriatr Soc. 2003;51:841–6. 12757573 10.1046/j.1365-2389.2003.51267.x

